# Long term clinical result of implant induced injury on the adjacent tooth

**DOI:** 10.1038/s41598-021-87062-9

**Published:** 2021-04-12

**Authors:** Yang-Jin Yi, In-Woo Park, Jeong-Kui Ku, Deuk-Won Jo, Jung-Suk Han, Young-Kyun Kim

**Affiliations:** 1 Department of Prosthodontics, Section of Dentistry, Seoul National University Bundang Hospital, 82, Gumi-ro 173 beon-gil, Bundang-gu, Seongnam, Korea; 2 Department of Dentistry and Dental Research Institute, School of Dentistry, Seoul National University, 101, Daehak-ro Jongno-gu, Seoul, Korea; 3 Department of Oral and Maxillofacial Radiology, College of Dentistry, Gangneung-Wonju National University, 7, Jukheon-gil, Gangwon-Do, Gangneung, Korea; 4 Department of Oral and Maxillofacial Surgery, Section of Dentistry, Seoul National University Bundang Hospital, 82, Gumi-ro 173 beon-gil, Bundang-gu, Seongnam, Korea; 5 Department of Oral and Maxillofacial Surgery, Section of Dentistry, Armed Forces Capital Hospital, 81, Saemaul-ro 117, Bundang-gu, Seongnam, Korea; 6 Department of Prosthodontics, School of Dentistry, Seoul National University, 101, Daehak-ro Jongno-gu, Seoul, Korea

**Keywords:** Clinical trial design, Risk factors

## Abstract

The purpose of the retrospective study was to investigate the long-term result of implant-induced injury on the adjacent tooth. The subjects of this retrospective study were patients who had received implants and had tooth injury; direct invasion of root (group I), root surface contact (group II), or < 1 mm distance of the implant from the root (group III). Clinical and pathological changes were periodically examined using radiographs and intra-oral examinations. Paired t-tests and chi-square tests were used to evaluate the implant stability quotient (ISQ) of implant and tooth complications, respectively (α = 0.05). A total of 32 implants and teeth in 28 patients were observed for average 122.7 (± 31.7, minimum 86) months. Seven teeth, three of which were subsequently extracted, needed root canal treatment. Finally, 90.6% of the injured teeth remained functional. Complications were significant and varied according to the group, with group I showing higher events than the others. The ISQs increased significantly. One implant in group I resulted in osseointegration failure. The implant survival rate was 96.9%. In conclusion, it was found even when a tooth is injured by an implant, immediate extraction is unnecessary, and the osseointegration of the invading implant is also predictable.

## Introduction

There are many complications related to dental implants, such as surgical, implant loss, bone loss, peri-implant soft tissue, and mechanical and esthetic/phonetic complications. Of these, surgical complications are hazardous to patients, causing hemorrhage, neurosensory disturbances, adjacent tooth damage, etc.^1^. Nerve damage or trauma to the adjacent tooth after implant placement, and implant damage/removal by tooth lesions have been reported by many clinical studies^2–6^. However, all these were case reports of 1-3 patients only. Data-based studies in the field of surgical complications are limited, especially on injuries to the adjacent tooth due to the incorrect placement of endosseous dental implants^7^. Even in animal studies, experimental designs for tooth injury using dental implants have rarely been shown, and have been used only to evaluate the effect of periradicular lesions on the osseointegration of existing implants^8^ and the feasibility of periodontal ligament generation on an implant surface^9^.

However, to determine the clinical prognosis of tooth-implant injury, reports of orthodontic miniscrew on the effect of tooth-screw injury can be cited. Orthodontic miniscrews have been used extensively based on the concept of absolute anchorage by the user-friendly easy placement of small-sized screw^10^. However, because they are blindly placed in the inter-radicular space, trauma to natural teeth can be a major cause of failure. Thus, some clinical studies on the prognosis of natural tooth roots invaded by miniscrews have been published. Clinical studies of root injuries showed positive results. A 5-year retrospective study Borah and Ashmead^11^ showed a very low incidence of root impingement (0.47%) by 2340 transfixation screws in 387 patients with facial fractures. The impinged teeth remained intact and did not require extraction. Another retrospective study reported 1.5% iatrogenic injury to roots during intermaxillary fixation for mandibular fractures. All scratched roots remained vital and root canal perforated teeth were treated endodontically later^12^. In a prospective study of patients with mandibular fractures, the incidence of clinically significant damage appeared to be low, although approximately 27.1 % of the 232 screws had damaged the roots^13^.

Histological changes after tooth injury have also been studied. In a prospective study of 68 teeth in 17 patients, Ahmed et al.^14^ intentionally injured premolars using a temporary skeletal anchorage device, scheduling them for extraction. They presented the repair process of injury in histological sections, showing that 70% of all premolars exhibited good cementum repair. The effect of the proximity of the screw to the root, the extent or process of healing according to the extent of damage or miniscrew existence, and the prognosis of the injured teeth and/or invading miniscrews have been studied well in animals such as dogs and minipigs^10,15–22^.

Despite clarity on the prognosis of tooth injury with a miniscrew, from animal studies, it remains unclear whether these results can be applied to dental implants invading adjacent human teeth^22^. Miniscrew differ from dental implant in terms of the insertion area, drilling path and load-bearing mechanism. The miniscrew penetrates the lateral surface of the bone and the root horizontally but dental implants are inserted longitudinally from the top of the crest bone. Since dental implants are placed parallel to the adjacent root surface, the injury mechanism from miniscrew cannot be extrapolated to that from endosseous dental implants.

Ribas et al.^7^ claimed that inadequate distance between the implant and the adjacent teeth was the most common problem during implant positioning. However, when an implant invades an adjacent tooth during surgery, the dentist has no evidence-based information to reverse the damage till now. As mentioned earlier, human case reports are not enough regarding the prognosis of teeth and implants. The long-term results of clinical studies on tooth injury by dental implants are also still unknown, except for Rubenstein and Taylor’s^23^ 10-year follow-up case report on patient with apical nerve transection. Thus it is not easy to determine whether to extract the damaged tooth or to remove invading implants.

The aim of this retrospective clinical study was to investigate the long-term result of implant-induced injury to the adjacent tooth. Therefore, accidental cases of adjacent root damage during implant insertion were gathered, and the clinical and pathological changes in both the tooth and the implant were examined longitudinally.

## Methods

This retrospective study was conducted under the approval of the Institutional Review Board of Seoul National University Bundang Hospital and independent ethics committees approved the protocol (IRB No. B-2012-655-103)., and each participant gave written, informed consent. The study was performed according to the Declaration of Helsinki and the requirements of Good Clinical Practice.

The subjects were patients who received implants at the Seoul National University Bundang Hospital, Section of Dentistry between October 2002 and September 2013. Digital panoramic radiographs of the subjects were taken at the time of oral examination, healing abutment/cover screw connection after surgery, and after loading serially. Digital periapical radiographs were also taken after surgery, at the time of superstructure connection, and at the periodic recall check-up. Radiographs were collected using a picture archiving and communication system (PACS) (INFINIT; Infinit, Seoul, Korea). Among patients, who had the implants that either injured the adjacent natural teeth or were placed with a distance of less than 1 mm from the adjacent root were selected for analysis. Patients with a follow-up record of at least one year's interval were finally included in the study.

Radiographs were taken by a single radiologic technologist, using the paralleling technique. In order to avoid false positive results due to angulation errors, the extent of implant-root contact was carefully checked by varying the x-ray taking angle when injury was noticed after surgery and during regular periodic check-ups. Tooth injury was confirmed by an oral and maxillofacial radiologist. Groups were classified according to the periapical view of the root region of the injured natural teeth: direct invasion of the root (group I), root surface contact (group II), and less than 1 mm distance of the implant from the root (group III). Considering a constant distance between the threads (thread pitch) of 0.6 mm to 0.9 mm, according to the system, the distance from the root surface to the implant was measured and calculated by the enlargement ratio in periapical radiographs.

The total number of implants was counted and the frequency of tooth injury was calculated. Records of the implant stability quotient (ISQ) using a magnetic resonance device (Osstell Mentor; Osstell, Gothenburg, Sweden) immediately after surgery (for primary stability) and at the second surgery or restoration (for secondary stability) were investigated to evaluate the stability change of implants after injury of adjacent tooth. Clinical and pathological changes of the injured teeth and the occurrence of complications were examined through clinical records and periodic periapical and/or panoramic views of the injured natural teeth and implants.

The paired t-test for ISQ change and the Chi-square test for the comparison of complications between groups were performed for statistical analysis (α=0.05) using SPSS 25 (SPSS, Chicago, USA).

### Ethical approval

The study was approved by the Institutional Review Board of Seoul National University Bundang Hospital (IRB No. B-2012–655-103).

## Results

A total number of 28 patients (16 male, 12 female, mean ages of 59.6 (±11) years) with 32 implants and injured adjacent natural teeth were included in this retrospective study. The mean postoperative follow-up was 122.7 (±31.7) months (minimum 86 months, maximum 184 months) (Table 1). The total number of implants placed was 3287. The frequency of injury by implants was less than 1%.

**Table 1:** Patients record of implant injury on the adjacent teeth.

No	Age (year)	Gender	Implant site	Implant System	Diameter (mm)	Length (mm)	Implant stability quotients		Adjacent tooth	Proximity	Tooth status		F/U (year)
							Primary	Secondary			Before	After	
1	56	Male	#14	Zimmer	4.1	10	69	70	#13	Proximity	Vital	NS	8.0
2	54	Male	#14	Zimmer	4.1	10	76	73	#13	Invasion	RCT	Prior RCT	8.2
3	71	Male	#24	Biohorizon	4	13			#23	Contact	RCT	Prior RCT	14.1
4	65	Male	#24	Osstem US	4	15			#23	Invasion	Vital	New RCT	14.9
			#14	Osstem GS III	4	10			#13	Contact	Vital	NS	11.8
5	23	Male	#15	Astra	4	11			#14	Contact	Vital	NS	9.8
6	63	Female	#15	Osstem GS III	4	11.5	60	80	#14	Contact	Vital	New RCT	9.6
			#46	Osstem GS III	4	10			#45	Contact	Vital	NS	9.6
7	66	Male	#36	Osstem GS II	5	13			#35	Invasion	Vital	Extraction	9.2
8	64	Male	#25	Dentium Implantium	3.4	10	55	77	#24	Contact	Vital	NS	8.3
9	68	Male	#25	Oneplant	4.3	11.5			#24	Contact	Vital	NS	12.9
10	60	Female	#16	ASTRA OsseoSpeed	4	11			#15	Proximity	Vital	NS	8.9
11	68	Female	#34	Osstem TS III	4.5	10	78	83	#33	Proximity	Apical lesion	Apical lesion	7.4
			#15	Osstem TS III	4	10			#14	Proximity	Vital	NS	7.2
12	55	Female	#34	Dentium Implantium	3.8	10	46	77	#33	Proximity	RCT	Prior RCT	11.1
13	57	Male	#14	Dentium Superline	3.8	12	72	72	#15	Contact	RCT	Prior RCT	8.6
14	46	Female	#35	Osstem GS III	4.5	10			#34	Invasion	Vital	New RCT	10.6
			#44	Osstem GS II	4	11.5	52	59	#43	Invasion	Vital	New RCT	11.7
15	73	Male	#36	Zimmer	4.7	8			#35	Proximity	Vital	NS	8.6
16	61	Male	#26	Osstem GS II	4.5	13	44	71	#25	Proximity	Vital	NS	7.3
17	65	Female	#34	Dentium Superline	4.5	8	53	63	#33	Invasion	Vital	NS	7.6
18	63	Female	#41	Osstem MS	2.5	13			#42	Contact	Vital	NS	7.7
19	60	Female	#14	3I Osseotite	4	13			#13	Invasion	Vital	NS	14.5
20	63	Female	#35	Neoplant Sinusquick	4	7	75	69	#34	Invasion	Vital	Extraction	7.8
21	62	Female	#36	Osstem GS II	4.5	7	71	82	#35	Invasion	RCT	Prior RCT	11.0
22	60	Female	#36	AVANA SSII	4.8	11.5			#35	Proximity	Vital	NS	13.9
23	44	Male	#37	Osstem TS III	5	8.5	65	72	#36	Invasion	Vital	NS	7.3
24	51	Male	#46	3-I Osseotite	4	10			#45	Proximity	Vital	Extraction	14.8
25	40	Male	#33	Osstem US II	3.3	10			#32	Contact	Vital	NS	15.3
26	71	Male	#36	Dentium Superline	6	10	82	86	#35	Proximity	Vital	NS	7.3
27	68	Male	#45	Neoplant SinusQuick	5	10			#44	Proximity	Vital	NS	11.0
28	73	Male	#16	Neoplant SinusQuick	5	11.5			#15	Proximity	Vital	NS	11.5

* FDI tooth numbering system.

F/U (follow-up), RCT (root canal treatment), Prior RCT (RCT prior to implant surgery), New RCT (RCT after surgery due to apical lesion or symptoms onset), NS (non-specific change).

The injured natural teeth and the corresponding implant placement sites are listed in Table 2. Damages to the natural teeth was most common in the maxillary canines and mandibular second premolars. Of the injured natural teeth, four were found to have root curvature. At the moment of implant placement, 15 of 32 damaged teeth had unnatural coronal contours with provisional/artificial crowns or loss of coronal structure.

**Table 2:** Location and incidence of affected tooth/implant.

	Maxilla		Mandible	
	Tooth	Implant	Tooth	Implant
Central incisor				1
Lateral incisor			2	
Canine	6		4	1
First premolar	5	7	3	4
Second premolar	4	5	7	3
First molar		3	1	7
Second molar				1
Total	15		17	

The ISQ of 14 out of the 32 implants was recorded (Table 1). Some records could not be completed because (1) surgery had been performed before the acquisition of the device, or (2) one of two stability ISQs was missing. The measured mean primary stability (ISQ) was 64.1 (±12.4). The mean secondary stability (ISQ) was 73.9 (±7.6), indicating a statistically significant increase in ISQ during the healing period (paired t-test; P=0.007).

**Figure 1:**
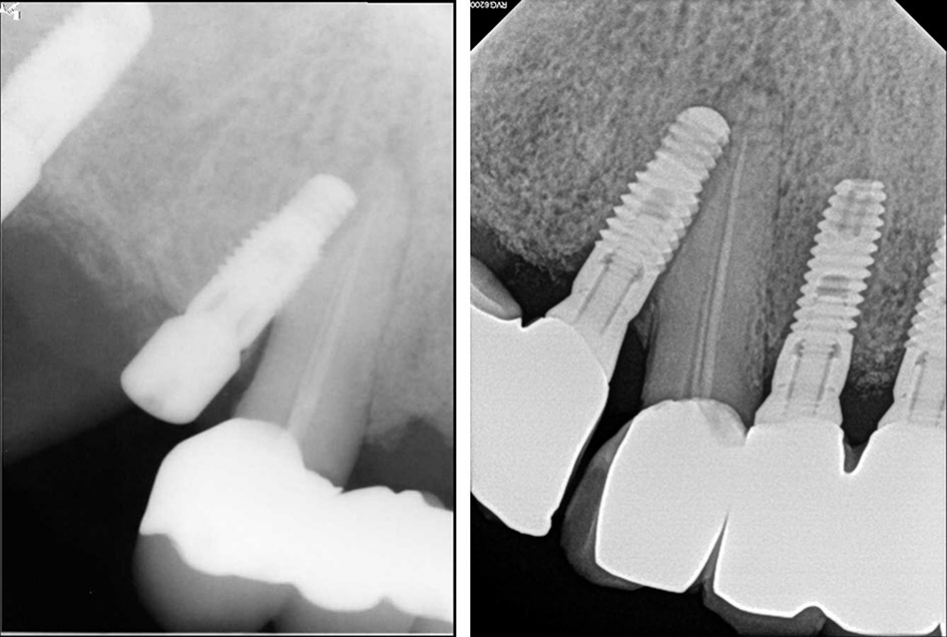
Direct invasion of the endodontic treated adjacent tooth by an implant. (Left) At the time of implant surgery, June 2012; (right) at 8 years after injury, August 2020.

Among the natural teeth that were injured, five of 32 teeth had already undergone root canal treatment (RCT) prior to implant placement. Five of these non-vital teeth functioned well without unusual changes, regardless of the extent of invasion (Fig. 1, Table 3). Of the 27 non-treated teeth, seven had a new RCT after implant injury due to the presentation of clinical symptoms and/or signs. Three of the seven new RCT teeth were subsequently extracted after 39 months, 7 years, and 14 years of function, respectively. One of the seven new RCT teeth was intentionally replanted after endodontic treatment and external root resorption was observed later. Although apical radiolucency was found before surgery, one of the 27 teeth was maintained without symptoms. Subsequently, 19 of 27 teeth (70.4%) remained vital and had no abnormal response during follow-up.

**Table 3:** Complication frequency according to the group.

Teeth		Event	No event (Intact)
Group I	RCT prior to surgery	0	2
	Vital	4 new RCT (2 subsequent extraction), 1 intentional replantation after RCT	3
	Total	5	5
Group II	RCT prior to surgery	0	2
	Vital	1 new RCT	7
	Total	1	9
Group III	RCT prior to surgery	0	1 (+ 1 apical radiolucency)
	Vital	1 new RCT (1 subsequent extraction)	9
	Total	1	11

RCT (root canal treatment), Prior RCT (RCT prior to implant surgery), New RCT (RCT after surgery due to apical lesion or symptoms onset).

**Figure 2:**
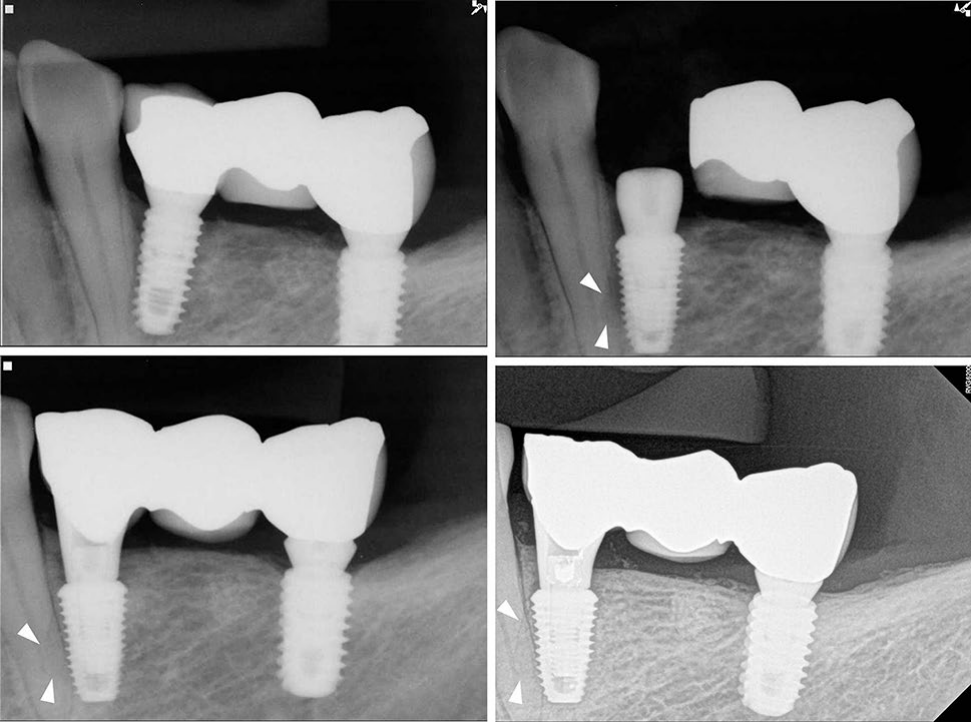
Direct invasion of the root (Group I); (left above) January 2013. (Right above) Osseointegration failed and a new implant was replaced. Arrows indicate trace of the old implant outline, February 2013; (left below) May 2013; (right below) injured tooth remained vital during 7-year functions, September 2020.

**Figure 3:**
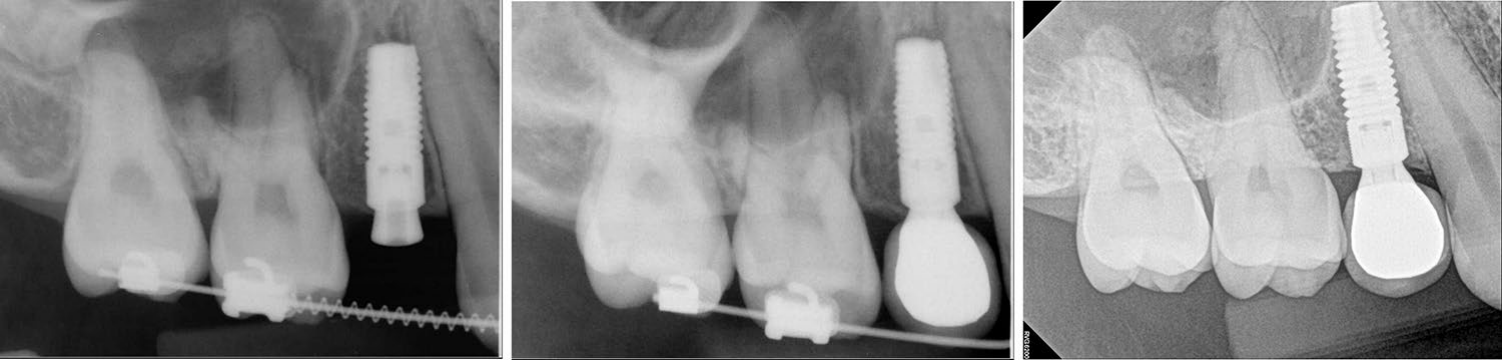
Root surface contact (Group II); (left) At the time of implant surgery, July 2010; (center) December 2010; (right) tooth remained vital during 10-year functions, May 2020.

**Figure 4:**
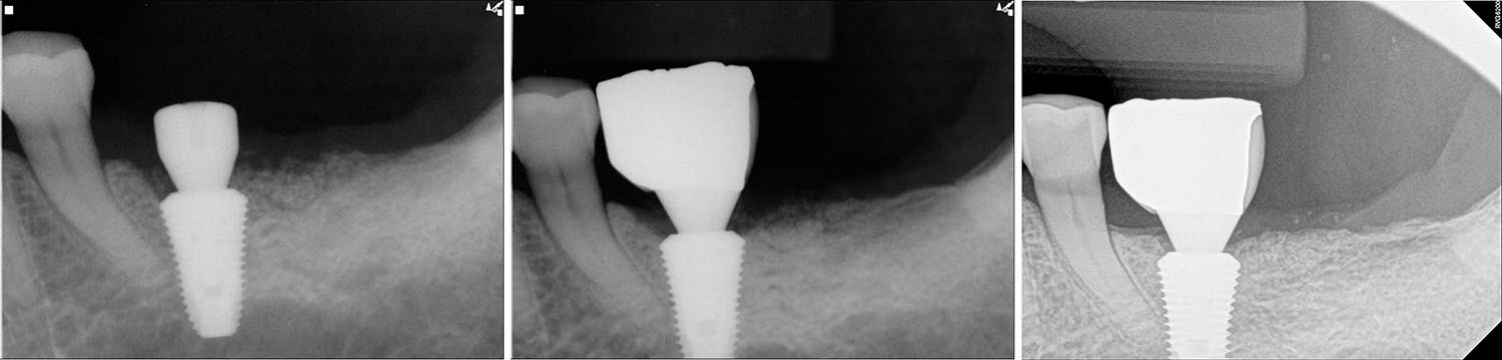
Less than 1 mm distance from the root (Group III); (left) At the time of implant surgery, February 2013; (center) December 2013; (right) tooth remained vital during 7-year functions, May 2020.

The periapical views of groups I, II and III are shown in Figs. 2, 3, 4. Of the 32 teeth, ten were in group I, ten in group II, and 12 in group III (Table 3). In group I, two out of five new endodontically treated teeth were extracted with symptoms. Consequently, five of 10 teeth in group I had events. The remaining five teeth consisted of two endodontically treated teeth prior to implant surgery and three vital teeth with non-specific findings. Fig. 2 shows the healing aspect of one of three vital teeth in group I and a replaced implant with a new one due to failure in osseointegration, which was the only case of implant failure in the study. In group II, one of the vital teeth had symptoms and underwent RCT. In group III, a new RCT was performed on one vital tooth, which was subsequently extracted. There were no implant failures in groups II and III. Events among the three groups showed significant differences (Chi-square test, p=0.008).

The patients complained only of a few clinical symptoms, even when the implant injured the adjacent natural teeth. Four patients complained of symptoms, and three of them were belonging to group I (directly invasion by the implant). Immediately after surgery, one patient with the implant in the maxillary right first premolar area complained of tenderness on percussion of the maxillary right canine, which had already been endodontically treated. However, the symptom disappeared after several days. Another patient in group II complained of tooth discomfort after surgery and underwent RCT with signs of pulp necrosis. Yet another patient complained of minute tooth discomfort after surgery, and intentional replantation was performed five years later. The last patient reported intermittent inconvenience during mastication with the tooth which had undergone RCT one month after loading. Eventually, the tooth was extracted three years and three months later with discomfort.

New RCT (seven teeth) and subsequent extraction (three of the seven new RCT teeth) were performed for 32 injured teeth (21.9%). With three teeth being extracted, the remaining 29 teeth functioned well, showing a survival rate of 90.6% for the injured natural teeth. Of the 32 implants, one failed and was replaced with a new implant. The survival rate of the implants was 96.9 %.

## Discussion

This study was conducted to investigate the clinical and pathological result of tooth injury without removing of the implant or the tooth. To the best of our knowledge, this is the first study dealing with the long-term results of dental implants invading and accidentally injuring the adjacent teeth.

The survival rates of implants were very high over at least the 7-year function evaluated in this study. There were only seven events (RCT and extraction) noted in 32 teeth, and only three teeth were eventually extracted from seven newly endodonticcally treated teeth. There was also a low incidence rate of complications beyond expectations. This is in agreement with the results of previous studies^13,23^. In one case report, the authors showed a stable bone level of the implant after 10 years of implant invasion onto the adjacent premolar root apex^23^. Also, after RCT, the tooth was almost asymptomatic and well-healed. In other clinical studies, infection was surprisingly uncommon although it was from the result of miniscrew^11^, and most of teeth usually remained vital with normal mobility throughout follow-up^6,11–14^.

In the present study, only four patients with direct invasion of the implant reported clinical symptoms after damage. The clinical symptoms were also non-specific from root injury in previous studies. Although some discomfort or pain was anticipated from damage to the root, human studies claimed that there was non-specific clinical symptoms^11–13^. In their prospective study, Ahmed et al.^14^ reported that no subject complained of undue pain after the trauma. Yoon et al.^6^ also reported no clinical symptoms in their case report. Rubenstein and Taylor^23^ described that although a patient reported mild discomfort after eating and paresthesia of the lower lip, nerve transection did not result in acute symptoms after implant placement; the symptoms were mild, transient, and reversible. In contrast, Margelos and Verdelis^2^ reported clinical symptoms of patient and necrosis of teeth next to the implant sites. The difference could be related to infection or inflammation of the injured sites.

Normal function of the injured teeth indicated healing after trauma from the implant. In Fig. 2, a trace of the implant form in the damaged root is seen even after replacement with the new implant. The trace remained for 7 years, and the radiolucent outline of the implant shape was associated with periodontal ligament regeneration. This regeneration proves the results of animal studies on the healing process of injured root surfaces with miniscrews. Hembree et al.^17^ showed that the periodontal ligament recovered to normal widths when miniscrew placement displaced the bone into the periodontal ligament. Kang et al.^18^ also reported the repair of moderately injured roots with osteoid and/or cementoid tissues with a normal periodontal ligament. The root cementum or periodontal ligament regenerated in 2-3 weeks after damage^22^.

In the present study, the teeth were divided into three groups, and group III (distance of less than 1 mm between the implant and the root surface) was designated based on the results of previous studies, in which the root damage developed even when the miniscrew was close to the root^10,22^. Kim et al.^10^ showed that root resorption was evident if the miniscrew was placed less than 1 mm from the periodontal ligament space. They concluded that at least 1 mm between the root surface and miniscrew was required to avoid complications.

In the result of present study, there was a significant difference in complication rates between the groups. When compared with groups II or III, group I showed relatively higher cases of pulp devitalization (five of 10 teeth) and progressive mobility for some years after damage. When invasion was limited to the periodontal ligament or was close to the root surface, the incidence of events was lower, but direct invasion of the root resulted in a higher rate of loss of vitality and/or subsequent extraction. Previous studies have shown that healing does not occur to the same extent in all cases. The pattern of repair differs according to the magnitude and nature of damage^17,18,20–22^. Minimally damaged roots did not adversely affect the healing process^18^. Conversely, if the pulp was invaded by the miniscrew, little or no healing occurred^17,21^.

We also determined whether the maintenance of implant contact with the root surface negatively affected the healing of the root. Hembree et al.^17^ concluded that short- and long-term healing was evident with unloaded miniscrews that remained in contact with the roots, though varying degrees of healing were noted. However, most of studies have reported that root contact with the miniscrew was the main reason for the disturbance of healing^10,15,19,22^. Kim et al.^10^ from their minipig study, concluded that immediate removal of the miniscrew leads to cementum repair, whereas leaving the miniscrew in place causes either delayed repair or no repair at all. No definite cementum repair was observed on the damaged root surface when any part of the thread was left touching the root surface. Chen et al.^19^ observed that most of the surrounding tissues were soft tissue with little direct bone-screw contact, if the miniscrews were kept in the damaged root sites. They highly recommended removal of screw, when miniscrew was found to be in contact with adjacent root. A systematic review of literature recommended the quick removal of the screws when the miniscrew was found to be in contact with the adjacent root, in order to avoid human/ animal root resorption and to regenerate the healing process^22^. In the present study, besides three teeth, the teeth invaded by dental implants functioned well during the long-term follow-up. This implies that most roots healed naturally without the need for implant removal even when they were in contact with implants. Thus, most of the invaded teeth may survive even without the removal of the implant, although some healing process might be disturbed.

From the viewpoint of implant stability, possibility of osseointegration without removal may be questionable when the implant contacted or invaded the adjacent root. In previous studies with miniscrews, miniscrew-root contact may cause the loss of miniscrew stability, which may be a major risk factor for failure^16,18,19,22^. In a study of beagle dogs, six screws were in contact with the root and five of them were lost. Conversely, as long as no contact was present between the root and miniscrew and the success rate was 100%^16^. In a similar study, out of 30 miniscrews with root contact, 25 failed, but 18 without contact, 8 failed only^19^. In another study, the failure rate of the miniscrews that invaded the roots was 79.2%, regardless of force application. This could be due to insufficient bone-implant contact for mechanical retention, damage to the surrounding tissue from miniscrew slip during insertion, and physiologic movement of the root during mastication^18^. In the present study, the ISQ of the measured implants increased significantly and osseointegration was not disturbed. Implant osseointegration and normal function continued for a long period. Even in group I, in which a relatively high failure rate of the invaded teeth was observed, of the 10 implants, only one resulted in the loss of osseointegration, and the remaining showed a stable bone level without pathological signs. This was the opposite results of previous animal studies using miniscrews. Considering the physiologic movement of invaded teeth during healing, dental implants showed higher stability than miniscrews. Dental implants could be maintained without immediate removal even when they invade the adjacent root.

In a study of periodontal healing following experimental injury to the root surface of human teeth, Hellden^24^ showed the vitality of the tooth was of limited importance for the healing of injury to the root surface. There were no failures in the injured teeth with RCT prior to implant surgery in this study. In contrast, of the teeth endodontically treated after surgery, three cases in this study resulted in extraction, but it was not clear that that was related to the moment of RCT. One case showed signs of ankylosis^25,26^ and external root resorption originating from intentional replantation.

Another tooth showed asymptomatic apical radiolucency but was maintained without RCT in this study. The effect of apical lesions from adjacent teeth on implant integration shows controversial results^3–5,8^. Shabahang et al.^8^ concluded that teeth with periapical lesions do not adversely affect adjacent implants in the four groups of dog studies. The groups were (1) no lesion/no RCT, (2) lesion/RCT, (3) lesion/RCT + implant detoxification, and (4) lesion/no RCT. The average integration rates of the apical part of the implant was 54%, 74%, 56% and 68%, respectively, without a significant difference. The formation of lesions adjacent to previously placed implants did not harm the affected tooth or the implant surface, even in the absence of treatment. However, a case report has shown that periapical pathology contaminates the implant and inhibits osseointegration of the implant during healing^3^. As previously mentioned, infection was very rare but the contamination of dental implants may still occur following endodontic treatment.

To avoid accidental injury to adjacent natural teeth by implants, some factors have been suggested. Root damage could be due to insufficient analysis of radiographic or study models, inappropriate insertion (low proximity and/or wrong angle, wide implant), and individual root shape variations such as abnormal root curvature or the distally tilted axis of the maxillary canine^4,6,22^. Other authors have reported firm tactile sensation and increased torque during miniscrew drilling when the root comes in contact with the miniscrew^19,21^. In the present study, the 15 injured teeth had either lost the coronal part or were either prepared or restored artificially. Four teeth were found to have root curvature. As these could cause confusion regarding the tooth axis, an incorrect drilling angle could occur with novice implant surgery. In recent years, guided surgery based on cone beam computerized tomography (CBCT) planning using digital modalities has been developed and is a good option for the correct placement of implants, even in unexpected conditions of limited space.

The limitation of this study was that root invasions were confirmed by two-dimensional periapical or panoramic views. Two-dimensional radiographs can cause misconceptions, although the angle was varied to examine the exact distance between the implant and the adjacent tooth. This limitation has also been criticized in a previous study by Fabbroni et al.^13^, who indicated that radiographic assessment overstates the frequency of root contact where there is none. In another study, histology showed that the damage did not occur in 26.2% of cases despite radiographs indicating damage contact^17^. Similarly, specimens grouped by radiographs were regrouped by tissue sections in another study^19^. Some studies have shown that CBCT is a helpful modality for implant studies to avoid misinterpretation of implant placement status^7,20,27^. Lv et al.^20^ measured the effective damage rate of roots through CBCT examination in their miniscrew study. Du Toit et al.^27^ stressed the use of CBCT for implant case preparation and planning to minimize nerve injury with their case series of inferior alveolar nerve injury. In the future study, CBCT would be required for proper evaluation of the region and extent of damage by implants.

## Conclusion

Within the limitations of this clinical and radiographic retrospective study, it can be concluded that even when a tooth is injured by an implant, immediate tooth extraction is unnecessary. The Osseointegration of the invading implant is also predictable. Although meticulous planning and surgery for implant placement should be a primary concern to avoid accidental injury to adjacent teeth, the good prognosis of teeth in this study shows that a conservative approach of the affected tooth/implant is also reliable.

## Abbreviations

ISQs: Implant stability quotients
RCT: Root canal treatment
